# Ceritinib in Japanese patients with anaplastic lymphoma kinase (*ALK*)+ non-small cell lung cancer: interim analysis results of a post-marketing surveillance study

**DOI:** 10.1038/s41598-020-72863-1

**Published:** 2020-10-08

**Authors:** Hiroyasu Kaneda, Minako Kizaki, Masami Ochi, Naoko Shiraiwa, Shigemi Akatsu

**Affiliations:** 1 Osaka City University, Osaka, Japan; 2 Novartis Pharma K.K., Tokyo, Japan

**Keywords:** Cancer, Oncology

## Abstract

Ceritinib is a selective anaplastic lymphoma kinase (ALK) inhibitor approved for the treatment of patients with unresectable advanced and/or recurrent *ALK* fusion gene-positive non-small cell lung cancer (NSCLC). As per the approval condition in Japan, this post-marketing surveillance (PMS) study evaluated the clinical safety (including adverse events [AEs], adverse drug reactions [ADRs] and priority investigation items) and efficacy (including ORR and PFS) of ceritinib in Japanese patients. Interim analysis was conducted ~ 2 years after the start of this non-interventional, multicentre, uncontrolled, open-label, special drug-use investigation and results are reported from March 28, 2016 to April 28, 2018. Each patient was followed up for 1 year. Most patients started treatment with 750 mg ceritinib. Safety profile was similar to that observed at the time of approval. No new AEs or ADRs with incidences higher than that at approval were identified. The rate of gastrointestinal ADRs (nausea, vomiting and diarrhoea) was 73.64%. Meaningful efficacy was observed in both post-crizotinib and post-alectinib settings, with ORR of 29.55% (95% CI 20.29–40.22) and disease control rate of 53.41% (95% CI 42.46–64.12). No concerns regarding the safety and efficacy of ceritinib were identified. No new measures, including modification of the PMS study protocol, are considered necessary.

## Introduction

Ceritinib is an oral anaplastic lymphoma kinase (ALK) inhibitor approved for the treatment of *ALK*+ non-small cell lung cancer (NSCLC) in the United States^1^ and the European Union^2^. In Japan, ceritinib was initially approved for crizotinib-resistant or intolerant *ALK*+ unresectable advanced/recurrent NSCLC (March 2016), and later received supplemental approval for *ALK*+ unresectable advanced/recurrent NSCLC (September 2017)^3^. Considering the limited number of Japanese patients enrolled in the clinical trials assessing the safety and efficacy of ceritinib, one of the conditions for approval of ceritinib in Japan was to conduct a post-marketing surveillance (PMS) study as part of the risk management plan in all patients treated with ceritinib.

The objective of this study was to evaluate the clinical safety and efficacy of ceritinib in Japanese patients with *ALK*+ unresectable advanced/recurrent NSCLC and also to identify any safety and efficacy issues in paediatric, elderly and pregnant patients and in patients with renal/hepatic impairment, interstitial lung disease or heart disease, if they were enrolled in the study.

## Results

**Figure 1:**
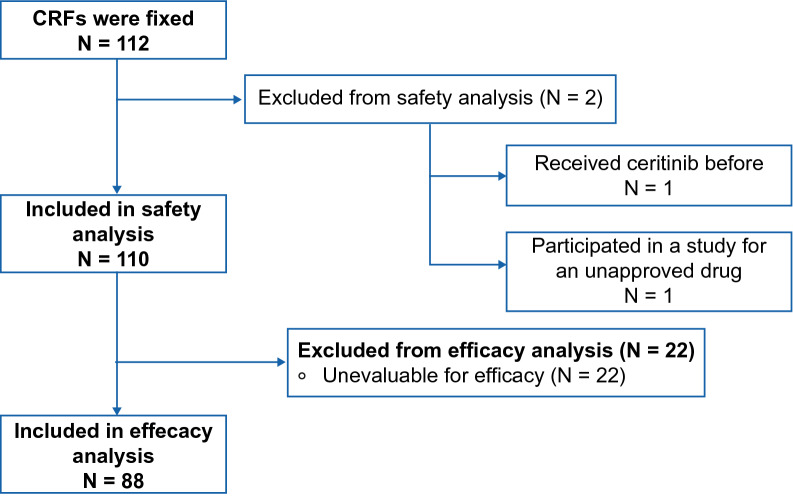
Patient composition. *CRF* case report form.

During the period from March 28, 2016 to April 28, 2018 (data cutoff date), 112 case report forms (CRFs) from 80 sites were collected, and 110 patients were included in the safety analysis population. Of these 110 patients, 88 were included in the efficacy analysis population (Fig. 1).

### Baseline characteristics

The baseline patient characteristics are presented in Table 1. Of the 110 patients included in the safety analysis, 46 patients (41.82%) were aged ≥ 65 years and 11 patients (10.00%) were aged ≥ 75 years. Patients below 18 years of age or pregnant patients were not treated with ceritinib. Sixty-three patients (57.27%) had brain metastasis at baseline. Majority of the patients had an Eastern Cooperative Oncology Group performance status (ECOG PS) of 0 (30%, n = 33) or 1 (47.27%, n = 52).

**Table 1:** Baseline patient characteristics.

Characteristic	N = 110
**Sex, n (%)**	
Male	51 (46.36)
Female	59 (53.64)
**Age, n (%)**	
< 18 years	0
18 to < 65 years	64 (58.18)
≥ 65 years	46 (41.82)
≥ 75 years	11 (10.00)
**Median age (range), years**	63.0 (29–82)
**ECOG PS, n (%)**	
0	33 (30.00)
1	52 (47.27)
2	12 (10.91)
3	11 (10.00)
4	2 (1.82)
**Lung cancer stage, n (%)**	
IIIB	10 (9.09)
IVIV	96 (87.27)
Unknown/not recorded	4 (3.64)
**Brain metastasis, n (%)**	
Absent	45 (40.91)
Present	63 (57.27)
Unknown/not recorded	2 (1.82)
**No. of prior lines of therapy, n (%)**	
0	0
1	3 (2.73)
2	13 (11.82)
3	38 (34.55)
≥ 4	56 (50.91)
**Prior therapies**^**a**^**, n (%)**	
Crizotinib	104 (94.55)
Alectinib	105 (95.45)
Combination chemotherapy including a platinum-based drug (e.g., platinum doublet)	88 (80.00)
Immunotherapy	7 (6.36)
Other chemotherapy	28 (25.45)

*ECOG PS* Eastern Cooperative Oncology Group performance status.

^a^Duplicate counting.

Of the 110 patients, 56 patients (50.91%) had received ≥ 4 prior lines of therapy and 38 patients (34.55%) had received 3 prior lines of therapy before starting treatment with ceritinib. Overall, 104 patients (94.55%) had received crizotinib as prior therapy, 105 patients (95.45%) had received alectinib and 88 patients (80.00%) had received combination chemotherapy including a platinum-based drug.

### Treatment

#### Dose and duration of treatment

The initial dose of ceritinib was 750 mg in 87 patients (79.09%), 600 mg in 15 patients (13.64%), 450 mg in 5 patients (4.55%), 300 mg in 1 patient (0.91%) and 150 mg in 2 patients (1.82%); administration was started from 750 mg (as the approved dosage of ceritinib at the data cutoff date) in most patients. The median value for the mean daily dose of ceritinib (Table 2) was 619.57 mg (range: 261.9–750).

**Table 2:** Mean daily dose of ceritinib.

Mean daily dose of ceritinib	n (%)
≤ 300 mg	2 (1.82)
> 300 mg to ≤ 450 mg	11 (10.00)
> 450 mg to ≤ 600 mg	36 (32.73)
> 600 mg to ≤ 750 mg	61 (55.45)
> 750 mg	0 (0.00)

The median duration of ceritinib use was 60.5 days (range: 1–364); duration of ceritinib use was calculated as day of the last dose − day of the initial dose + 1 (not considering the interruption period). The median value for the actual treatment duration of ceritinib use was 51.5 days (range: 1–364), where actual treatment duration with ceritinib was calculated as day of the last dose − day of the initial dose + 1 (excluding the interruption period).

#### Discontinuation

Of the 110 patients included in the safety analysis population, 6 patients (5.45%) completed the 52-week observation period, 95 patients (86.36%) discontinued or dropped out and 59 patients (53.64%) discontinued the treatment due to worsening of underlying disease.

### Safety

#### Adverse events and adverse drug reactions

Of the 110 patients in the safety analysis population, adverse events were noted in 107 patients (97.27%). A causal relationship with ceritinib was not ruled out in 99 patients, and these events were assessed as adverse drug reactions. The adverse drug reactions noted in ≥ 10% of patients (any grade) were diarrhoea (51.82%), nausea (36.36%), vomiting (28.18%), decreased appetite (15.45%), abnormal hepatic function (14.55%) and renal impairment (10%).

The most common grade ≥ 3 adverse drug reactions reported in at least 2 patients were diarrhoea (6.36%), nausea (5.45%), decreased appetite (3.64%), vomiting, abnormal hepatic function, liver disorder (each 2.73%), interstitial lung disease, drug-induced liver injury, increased alanine aminotransferase (ALT) and decreased platelet count (each 1.82%). The most common serious adverse drug reactions in at least 2 patients (excluding events related to the aggravation of the underlying diseases, such as stage IV NSCLC and malignant neoplasm progression) were nausea (2.73%), decreased appetite (1.82%) and interstitial lung disease (1.82%).

Adverse events leading to discontinuation of ceritinib were noted in 70 of 110 patients (63.64%). Adverse reactions leading to ceritinib discontinuation were seen in 33 patients (30%).

The most common adverse drug reactions leading to discontinuation of ceritinib in at least 3 patients (excluding events related to the aggravation of the underlying diseases, such as stage IV NSCLC and malignant neoplasm progression) were nausea, vomiting, liver dysfunction (each 4.55%) and renal impairment (2.73%).

#### Priority investigation items

Tables 3 and 4 provide details on the incidence of adverse drug reactions included in the priority investigation items.

**Table 3:** Incidence of adverse drug reactions included in priority investigation items.

Priority investigation item Preferred term	Safety analysis population (N = 110)	
	ADR any grade, n (%)	ADR grade ≥ 3, n (%)
**Total**	94 (85.45)	27 (24.55)
**Nausea/vomiting/diarrhoea**	81 (73.64)	15 (13.64)
Diarrhoea	57 (51.82)	7 (6.36)
Nausea	40 (36.36)	6 (5.45)
Vomiting	31 (28.18)	3 (2.73)
**Hepatic impairment**	37 (33.64)	10 (9.09)
Abnormal hepatic function	16 (14.55)	3 (2.73)
Increased alanine aminotransferase	7 (6.36)	2 (1.82)
Increased aspartate aminotransferase	7 (6.36)	0
Liver disorder	5 (4.55)	3 (2.73)
Drug-induced liver injury	2 (1.82)	2 (1.82)
Increased gamma-glutamyltransferase	2 (1.82)	0
Hepatic enzyme increased	2 (1.82)	0
Increased blood bilirubin	1 (0.91)	0
Increased transaminases	1 (0.91)	0
**Interstitial lung disease**	2 (1.82)	2 (1.82)
**Pancreatitis**	2 (1.82)	1 (0.91)
Increased amylase	1 (0.91)	0
Increased lipase	1 (0.91)	0
Hyperamylasaemia	1 (0.91)	1 (0.91)
**Infection**	2 (1.82)	1 (0.91)
Otitis externa	1 (0.91)	0
Pneumonia	1 (0.91)	1 (0.91)
**QT prolongation**	1 (0.91)	0
**Bradycardia**	1 (0.91)	0
**Pericarditis**	1 (0.91)	1 (0.91)
**Hyperglycaemia (including diabetes mellitus)**	0	0

*ADR* adverse drug reaction.

**Table 4:** Time to occurrence and time from occurrence to resolution of priority investigation items.

Priority investigation item	Time to ADR occurrence^a^		Time from ADR occurrence to recovery/remission^b^	
	n	Median (range), days	n	Median (range), days
Nausea/vomiting/diarrhoea	79^c^	3.0 (1–129)	69	23.0 (2–241)
Hepatic impairment	37	29.0 (3–238)	33	30.0 (8–204)
Interstitial lung disease	2	20.0 (14–26)	1	8.0 (8–8)

^a^Number of days from initial administration to first event onset (calculated as [event onset date] minus [initial administration date] plus 1 day).

^b^Number of days until the first onset event reaches recovery or remission outcome ([recovery or remission date] minus [onset date] plus 1 day).

^c^Two patients without data regarding the date of occurrence were excluded.

ADR: adverse drug reaction.

Of the 110 patients in the safety analysis population, nausea/vomiting/diarrhoea (73.64%) were the most common adverse drug reactions in the priority investigation items.

#### Safety in patients with special demographic characteristics

Adverse drug reactions were noted in 43 of 46 patients aged ≥ 65 years (93.48%), which included diarrhoea in 23 patients (50.00%), vomiting in 14 patients (30.43%), nausea in 13 patients (28.26%), decreased appetite and hepatic function abnormal in 10 patients each (21.74%) and increased blood creatinine in 9 patients (19.57%); gastrointestinal disorders were frequently noted. Serious adverse drug reactions were noted in 7 patients (15.22%), which included decreased appetite, dehydration, cerebral infarction, myocardial infarction, pneumonia aspiration, nausea, cholecystitis acute, liver disorder, pyrexia, increased ALT, increased gamma-glutamyltransferase and increased blood alkaline phosphatase in 1 patient each (2.17%).

Of the 11 patients aged ≥ 75 years, 10 patients (90.91%) experienced adverse drug reactions, including diarrhoea in 6 patients (54.55%) and increased blood creatinine in 3 patients (27.27%). Serious adverse drug reactions were noted in 2 patients (18.18%) and included aspiration pneumonia, acute cholecystitis, pyrexia, increased gamma-glutamyltransferase and increased blood alkaline phosphatase, each in 1 patient (9.09%).

Adverse drug reactions were noted in all 6 patients who had renal impairment while starting the treatment with ceritinib. These included renal impairment in 5 patients (83.33%), diarrhoea in 4 patients (66.67%) and vomiting in 2 patients (33.33%). Serious adverse drug reactions were interstitial lung disease, pneumonia and pulmonary alveolar haemorrhage that occurred in the same patient.

Of the 5 patients who had hepatic impairment while starting treatment with ceritinib, 3 experienced adverse drug reactions, which included each of interstitial lung disease, nausea, constipation, abnormal hepatic function and increased blood bilirubin in 1 patient (20.00%). A serious adverse drug reaction of grade 4 interstitial lung disease was noted in 1 patient.

Renal impairment, nausea, diarrhoea, interstitial lung disease, pneumonia and pulmonary alveolar haemorrhage were the adverse drug reactions noted in 1 patient who had interstitial lung disease while starting treatment with ceritinib; interstitial lung disease, pneumonia and pulmonary alveolar haemorrhage were of grade 3 or higher severity.

Adverse drug reactions were noted in 2 of 3 patients who had heart disease while starting treatment with ceritinib. Adverse drug reactions were diarrhoea and nausea in 2 patients (66.67%), and each of pneumonia, interstitial lung disease, pulmonary alveolar haemorrhage, vomiting and renal impairment in 1 patient (33.33%). Serious adverse drug reactions (interstitial lung disease, pneumonia and pulmonary alveolar haemorrhage) were noted in 1 patient.

#### Deaths

Of the 110 patients in the safety analysis population, 29 patients (26.36%) died. Adverse drug reactions were noted in 4 patients. The primary causes of death were malignant neoplasm progression in 27 patients (24.55%), stage IV NSCLC in 21 patients (19.09%), NSCLC in 4 patients (3.64%), metastasis to the central nervous system in 3 patients (2.73%) and metastasis to the meninges in 2 patients (1.82%). Adverse drug reactions that led to death (excluding events related to the disease progression [malignant neoplasm progression and stage IV NSCLC]) were pneumonia and pulmonary alveolar haemorrhage in the same patient (age, 59 years; gender, female).

### Efficacy

#### Tumour response

In the efficacy analysis population (88 patients), the best overall response rate (ORR; complete response [CR] + partial response [PR]) was 29.55% (95% confidence interval [CI] 20.29–40.22), and the disease control rate (CR + PR + stable disease [SD]) was 53.41% (95% CI 42.46–64.12) (Table 5).

**Table 5:** Best overall response and disease control rate per RECIST v1.1.

	**N = 88**
**Best overall response rate (CR + PR), n (%) [95% CI]**	26 (29.55) [20.29–40.22]
CR	1 (1.14)
PR	25 (28.41)
SD	21 (23.86)
PD	36 (40.91)
Not evaluable	5 (5.68)
**Disease control rate (CR + PR + SD), n (%) [95% CI]**	47 (53.41) [42.46–64.12]

*CI* confidence interval; *CR* complete response; *PD* progressive disease; *PR* partial response; *RECIST* Response Evaluation Criteria in Solid Tumors; *SD* stable disease.

#### Progression-free survival

**Figure 2:**
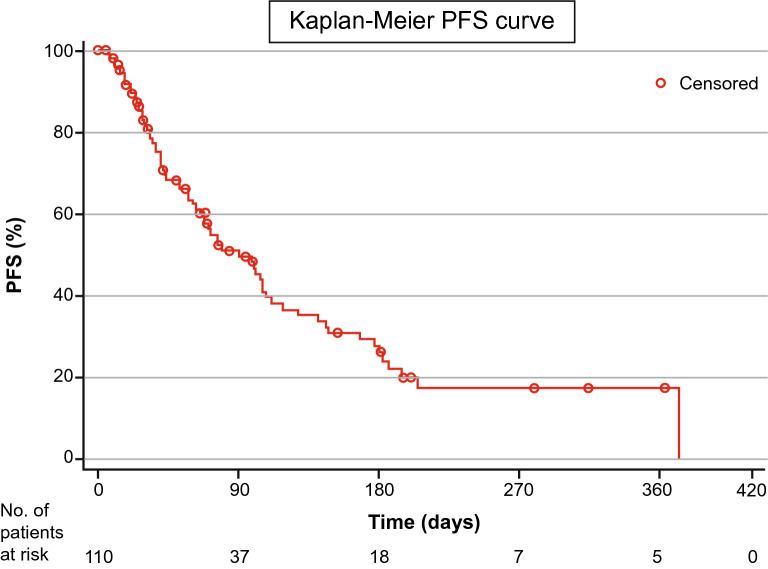
Kaplan–Meier curve for PFS. *PFS* progression-free survival.

Median progression-free survival (PFS; summarised for the safety analysis population with censored data on the end date of the safety analysis period) was 91.0 days (95% CI 66.00–112.00). Figure 2 depicts the Kaplan–Meier curve for PFS.

#### Tumour response per patient characteristics

Evaluation of the factors that may affect efficacy revealed that ECOG PS was the sole factor for which the 95% CI of the odds ratio within category did not include 1. While the response rate was 52.00% (13/25 patients) in ECOG PS “0” patients, it was 22.92% (11/48) in ECOG PS “1” patients; the 95% CI of the odds ratio did not include 1 (odds ratio: 0.27, 95% CI 0.10–0.77), and a significant difference was observed in the response rate. No significant difference was observed in other factors. Additional details regarding tumour response are included in Table 6.

**Table 6:** Best overall response per patient characteristics.

Factor	Category	N	Responders (CR + PR), N (%)	Odds ratio (95% CI)^a^
Efficacy analysis population		88	26 (29.55)	
ECOG performance status	0	25	13 (52.00)	–
	1	48	11 (22.92)	0.27 (0.10, 0.77)
	2	7	0	NE
	3	6	2 (33.33)	0.46 (0.07, 2.99)
	4	2	0	NE
Brain metastasis	Absent	35	11 (31.43)	–
	Present	52	15 (28.85)	0.88 (0.35, 2.25)
	Unknown/not reported	1	0	–
No. of prior lines of therapy	0	0	0	NE
	1	1	0	NE
	2	10	5 (50.00)	NE
	3	29	7 (24.14)	NE
	≥ 4	48	14 (29.17)	NE
Prior therapies	Crizotinib	86	26 (30.23)	–
	Alectinib	84	24 (28.57)	–
	Combination chemotherapy including a platinum-based drug (e.g., platinum doublet)	73	22 (30.14)	–
	Cancer immunotherapy	7	3 (42.86)	–
	Other chemotherapy	27	9 (33.33)	–
Age	< 75 years	80	23 (28.75)	–
	≥ 75 years	8	3 (37.50)	–
	< 65 years	52	13 (25.00)	–
	≥ 65 years	36	13 (36.11)	–

*CI* confidence interval; *CR* complete response; *ECOG* Eastern Cooperative Oncology Group; *NE* not estimable; *PR* partial response.

^a^Unknown/not recorded patients were excluded from the calculation of odds ratio.

## Discussion

This PMS study reported the results of the clinical use of ceritinib in patients with *ALK*+ unresectable advanced/recurrent NSCLC. The safety results reported in this PMS study are consistent with those reported in previous clinical studies^4,5^. Gastrointestinal disorders and hepatobiliary disorders were frequently noted, similar to the safety profile observed at approval.

For the priority investigation items, no issues requiring special attention were observed in this PMS study compared with the results of a pooled analysis of 6 clinical trials of ceritinib (studies X1101, X2101, A2201, A2203, A2301 and A2303; N = 822). QT prolongation was reported in 0.91% of patients in the PMS study versus 9.1% of patients in the pooled analysis. Interstitial lung disease was reported in 1.82% of patients in the PMS study versus 0.4% of patients in the pooled analysis. Hyperglycaemia was reported in 3% of patients and diabetes mellitus in 0.2% of patients in the pooled analysis, while no patient reported hyperglycaemia/diabetes mellitus in the PMS study. The incidence of nausea, vomiting and diarrhoea was lower in the PMS study compared with the pooled analysis: nausea, 36.36% versus 73.2%; vomiting, 28.18% versus 59.0%; diarrhoea, 51.82% versus 79.4%. This could be due to better management of gastrointestinal events with dose reductions at the appropriate time and administration of antiemetics or antidiarrhoeals. Pancreatitis was reported in 1.82% of patients in the PMS study (as a priority investigation item), while the pooled analysis reported pancreatitis in 0.1%, increased amylase in 5.8% and increased lipase in 4.6% of patients. Bradycardia was reported in 0.91% of patients in the PMS study versus 0.7% of patients in the pooled analysis. Pericarditis was reported in 0.91% of patients in the PMS study (as a priority investigation item), while the pooled analysis reported 1.7% of patients with pericarditis, along with 1.1% of patients with pericardial effusion. Infection was reported in only 1.82% of patients in the PMS study (pneumonia and otitis externa in 0.91% each), while the pooled analysis reported pneumonia in 1.3%, lung infection in 0.7%, oral candidiasis in 0.7%, gastroenteritis in 0.6% and pharyngitis in 0.5% of patients. Hepatic impairment was reported in 33.64% of patients in the PMS study (as a priority investigation item), while the pooled analysis reported up to 60% of patients with liver laboratory test abnormalities, 0.7% of patients with hepatotoxicity, 48.3% of patients with increased ALT and 39.5% of patients with increased aspartate aminotransferase (AST).

The safety profile reported in this PMS study is also in line with the safety profile observed in the Japanese subgroup of patients (N = 24) from the ASCEND-2 study (single-arm, open-label, multicentre, phase II study of ceritinib in adult patients with *ALK*+ NSCLC previously treated with chemotherapy and crizotinib)^6^. No new adverse events or adverse drug reactions with incidences higher than that at approval were identified in this study.

Clinically meaningful efficacy was observed with ceritinib in both post-crizotinib and post-alectinib settings. In the PMS study, the response rate was 29.55% (95% CI 20.29–40.22), and in the ASCEND-2 study, the response rate was 37.1% (95% CI 29.1–45.7) for the overall population and 45.8% (95% CI 25.6–67.2) for the Japanese subgroup (summary of the data until the first approval). It is important to note that the PMS study was less restrictive compared with clinical trials and did not set any patient inclusion/exclusion criteria in terms of performance status and previous therapy. The PMS study included a greater number of patients with a poor condition at baseline compared with the ASCEND-2 study, including patients who had an ECOG PS of 3 or higher, had brain metastasis at baseline or had received ≥ 4 prior lines of therapy. A direct comparison of the efficacy results was not feasible due to the difference in patient characteristics and also the limited tumour assessments in the PMS study.

There were no safety or efficacy issues in elderly patients and no safety issues among patients with renal/hepatic impairment or interstitial lung diseases/heart diseases.

As per the Clinical Practice Guideline for Lung Cancer in Japan (published on November 2019)^7^, ceritinib is recommended as the first-line therapy (PS 0–1) for patients with *ALK*+ NSCLC (recommendation grade 2B). Ceritinib as second- or later-line therapy is recommended for *ALK*+ NSCLC patients with resistance to or progression after treatment with first-line ALK-tyrosine kinase inhibitors (PS 0–2; recommendation grade 2C).

No new adverse events or adverse drug reactions whose incidences were especially higher than that at approval were identified and no new measures, including modification of the PMS study protocol, are considered necessary. The occurrences of adverse events and adverse drug reactions will continue to be monitored carefully, and efforts will be made to raise awareness among physicians and medical institutions by using the package inserts and other materials.

## Methods

### Patient population

As per the approval condition, this investigation aimed to include all Japanese patients treated with ceritinib for a specified post-marketing period. Patients starting ceritinib before the conclusion of the contract with the medical institution/municipality were allowed to enrol in the investigation and register after the conclusion of the contract.

### Study design

This multicentre, uncontrolled, observational study (specified drug-use surveillance; all-case) was conducted in accordance with the Good Post-marketing Study Practice (GPSP) ordinance in Japan. The study protocol and the implementation guideline (summary of the study protocol) were approved by a scientific/ethical review committee of Novartis. Then the implementation guideline was presented to and was approved at all participating sites/institutions by institutional review boards (IRBs) or approved according to each institutional rule. Informed consent was not obtained from the patients for this survey, because obtaining informed consent is not necessary in GPSP ordinance. However, as approved by all IRBs, before the survey was conducted, the following were explained to the patients by investigational doctors; "Objective and procedure of the survey", "Personal Information Protection Law" and "Publication of the survey information". Furthermore, none of the authors had access to identifying patient information when analysing the data because of Personal Information Protection Law. The names of the participating institutions are not listed in the manuscript due to restrictions on disclosure. The target sample size for registration was 520 patients with *ALK*+ unresectable advanced/recurrent NSCLC, and 500 patients were to be included in the safety analysis population. The target sample size for registration was set at 520 patients to make up for patients who might be excluded from the safety analysis population. A central registration system was adopted for patient registration. The data recorded by the investigator/sub-investigator in CRFs were collected for this investigation.

As this is a non-interventional investigation, visit schedule was as per routine medical practice. The investigator/sub-investigator recorded data in the CRFs at every patient visit, if possible.

Each patient was followed up for 1 year (52 weeks). Patients discontinuing or completing treatment with ceritinib in less than a year from the start of treatment were followed up to 30 days after the final dose, and the follow-up information was entered in the CRFs.

This study collected information including patient characteristics, data on ceritinib use (duration and dose), use of any concomitant drugs or therapies, laboratory parameters, pregnancy information in women, safety and efficacy.

### Safety assessments

Safety assessments included adverse events, adverse drug reactions (defined as an adverse event related to ceritinib), seriousness, priority investigation items (including hepatic impairment, QT interval prolongation, interstitial lung disease, hyperglycaemia [including diabetes mellitus], nausea/vomiting/diarrhoea, pancreatitis, bradycardia, pericarditis and infections) and laboratory parameters (blood biochemistry profile, including AST, ALT, total bilirubin, blood glucose, amylase, lipase, and electrocardiographic parameters, including QT interval and RR interval). Each priority investigation item included a specific group of preferred terms of standardised Medical Dictionary for Regulatory Activities (MedDRA) as per protocol. The severity of adverse events was evaluated as per the Common Terminology Criteria for Adverse Events (CTCAE) v4.0.

The safety analysis population included all patients whose CRFs were collected except those who met the following safety analysis exclusion criteria: patients whose CRFs were lacking the signature or name/seal of the investigator/sub-investigator, patients who received treatment out of the contract period, patients whose adverse events were not recorded, patients whose registration was not fixed, patients with duplicate registration, patients with prior ceritinib use, patients who had participated in a study for unapproved drugs, patients with off-label use and patients who had not visited the site after the initial dose.

### Efficacy assessments

Efficacy was assessed with ORR and PFS. ORR was evaluated as per the Response Evaluation Criteria in Solid Tumors (RECIST) v1.1 (using the best response at 6 months from the start of treatment or at the end of the observation period). Patients with confirmed CR or PR in the assessment were regarded as responders. ORR was assessed in the efficacy analysis population, which consisted of all patients included in the safety analysis population excluding those for whom an efficacy assessment was not feasible. PFS was evaluated from the start of treatment of ceritinib to the first onset of exacerbation or death and was assessed in the safety analysis population.

### Statistical analysis

The number and proportion of patients with onset of adverse drug reactions of priority investigation items were summarized by events. Odds ratio and its 95% confidence interval were estimated for categories in the analysis by patient characteristics. The data of "Unknown" and "Not described" were not included in the data for the odds ratio calculation. PFS was summarised using the Kaplan–Meier method. Statistical analysis were performed with SAS software, version 9.3, and adverse drug reactions were encoded using the MedDRA version 21.0 and classified according to their preferred terms.

## Data Availability

Novartis is committed to sharing with qualified external researchers access to patient-level data and supporting clinical documents from eligible studies. All data provided are anonymized to respect the privacy of patients who have participated in the trial in line with applicable laws and regulations.
